# Plant Growth and Soil Microbial Impacts of Enhancing Licorice With Inoculating Dark Septate Endophytes Under Drought Stress

**DOI:** 10.3389/fmicb.2019.02277

**Published:** 2019-10-09

**Authors:** Chao He, Wenquan Wang, Junling Hou

**Affiliations:** ^1^Institute of Medicinal Plant Development, Chinese Academy of Medical Sciences & Peking Union Medical College, Beijing, China; ^2^School of Chinese Pharmacy, Beijing University of Chinese Medicine, Beijing, China

**Keywords:** dark septate endophytes, plant performance, soil microbial community composition, drought, licorice, symbiosis

## Abstract

This study mainly aimed to investigate the effects of dark septate endophytes (DSE) (*Acrocalymma vagum*, *Paraboeremia putaminum*, and *Fusarium acuminatum*) on the growth and microbial community composition in the rhizosphere soil of a medicinal plant, licorice (*Glycyrrhiza uralensis*), grown in the non-sterile soil under drought stress. The results showed that three DSE strains could effectively colonize the plant roots and form a strain-dependent symbiosis with licorice. Although drought stress declined the growth of licorice plants, these decreases were partly recovered by DSE inoculation. Specifically, the inoculation of *A. vagum* and *P. putaminum* significantly increased the biomass and glycyrrhizin content, whereas *A. vagum* and *F. acuminatum* increased glycyrrhizic acid content of host plants under drought stress. However, the inoculation of *F. acuminatum* showed significant negative effects on the shoot, root, and total biomass of licorice plants. In addition, the effects of DSE inoculation on the morphological, photosynthetic, and antioxidant parameters of licorice plants, and mineral nutrient and microbial community composition in the rhizosphere soil were dependent on the DSE species as well as water regime. Interestingly, DSE inoculation significantly increased AM fungi content under drought stress. In addition, DSE associated with water had a significant positive influence on soil organic matter, available phosphorus (P), AM fungi, leaf number, soluble protein, SOD activity, total root length, root branch, and glycyrrhizic acid content. Based on the results of variance partitioning analysis, 17.0, 34.0, 14.9, 40.1, 28.2, and 18.0% variations in shoot morphology, root morphology, plant biomass, active ingredient, photosynthetic parameters, and antioxidant parameters, respectively, were attributable to the presence of certain soil microorganisms. These findings suggest the possibility that DSE inoculation improved the root development and nutrient absorption of host plants, altered the soil microbiota, and might also contribute to plant growth and survival under drought conditions. As *A. vagum* exhibited positive effects on the plant biomass, morphological and physiological parameters, and active ingredient content in licorice plants under drought stress, it was considered to be the best fungus for licorice cultivation. These results contribute to the understanding of the ecological function of DSE fungi in dryland agriculture.

## Introduction

Recently, because of human interference and global changes, the deficiency of water has negatively affected plant development and agricultural production in many regions worldwide, making it a major social and environmental problem (Singh et al., 2012; Bodner et al., 2015). Water deficiency inhibits root development, thereby decreasing the ability of plants to absorb minerals and water (Cochavi et al., 2019). However, plants have already developed different means for resisting a water deficit by altering their morphological and physiological properties (Asfaw et al., 2017; Li et al., 2017). Plants in natural habitats often harbor ubiquitous soil microbes, including some that are drought-tolerant and capable of stimulating plant growth (Xie et al., 2018; Lee et al., 2019). Plants provide important habitats and deliver photosynthates to their associated microbiome (Jiang et al., 2017; Santos-Medellín et al., 2017). In turn, plant growth and productivity were also determined from the soil-associated microbiome, which played an important role in nutrient availability and ecological functions (Bonkowski and Roy, 2005; Hol et al., 2010; Lau and Lennon, 2011). Several studies have shown that the adaptation of plant to stress habits is closely associated with the presence of soil microorganisms (Lata et al., 2018). For instance, the colonization of fungal endophytes is attributable to the tolerance of plants to high temperature, salt, and drought resistance (Rodriguez et al., 2008; Qiang et al., 2019). Concurrently, these associated fungal endophytes, including dark septate endophytes (DSE), have been shown to enhance plant productivity and health, and are therefore particularly beneficial for sustainable agriculture development (Aroca et al., 2008; Zhang et al., 2017).

Dark septate endophytes are conidial or sterile ascomycetous fungi that colonize the roots of mycorrhizal and non-mycorrhizal plants (Jumpponen and Trappe, 1998). DSE are characterized by their dark septate hyphae and melanized microsclerotia (Mandyam and Jumpponen, 2005; Xie et al., 2017). Related reports have indicated that the interactions between DSE and host plants vary from negative to neutral and positive (Newsham, 2011; Li X. et al., 2019). The use of DSE fungi as promoters can enhance plant growth by improving the mineral element uptake (Surono and Narisawa, 2017; Li et al., 2018; He et al., 2019), as well as by preventing the development of biotic and abiotic resistance in hosts (Surono and Narisawa, 2017; Li et al., 2018). Moreover, some studies have contributed our understanding regarding the fact that inoculation with DSE can also enhance the production levels of medical plant compounds (Wu et al., 2010; Zhu et al., 2015; He et al., 2019). In comparison to the well-studied arbuscular mycorrhizal (AM) fungi, little is known about the interactions between DSE and medical plants under drought conditions. In addition, rhizosphere soil is an intense field of microbial activity and plant stress responses (Mendes et al., 2013). Thus, rhizosphere-associated microbes play an important role in the decomposition of organic matter and nutrient maintenance, which had beneficial effects on the adaptation of host plants to different conditions (Dennis et al., 2010; Bai et al., 2015). Fiorentino et al. (2018) found that the composition of eukaryotic communities in the rhizosphere was more profoundly affected by the inoculation of *Trichoderma* in soils with a low nitrogen (N) concentration than in N fertilized soils. Lu et al. (2019) also indicated that inoculation with AM fungi could regulate the counterpoise of soil micrograms, and alter the components of root exudates, thus relieving the effects of replanting-related diseases. Despite the fact that the direct effects of beneficial microbial inoculants on plant growth and rhizosphere-associated microbes have been reported widely (Barrow and Aaltonen, 2001; Poosakkannu et al., 2017; Santos et al., 2017; Lata et al., 2018; He et al., 2019), little information is available regarding the contribution of DSE inoculation to the native rhizospheric microbial community.

*Glycyrrhiza uralensis* Fisch. (licorice) is a perennial leguminous species that is extensively distributed worldwide. Licorice was chosen as a host plant, mostly because of its important role in pharmacology and the restoration of vegetation (Hayashi and Sudo, 2009; Chen et al., 2017; Xie et al., 2018). Although studies have demonstrated that the symbiosis of AM with licorice plants under drought conditions could facilitate the growth and accumulation of secondary metabolites (Orujei et al., 2013; Xie et al., 2018), little is known about the influence of DSE either alone or in combination with drought stress on the growth, active ingredient accumulation, and soil microbial community of licorice plants. The results of our previous study showed that inoculation with DSE species (*Acrocalymma vagum*, *Paraboeremia putaminum*) enhances the growth of licorice plants grown in sterilized soil (He et al., 2019). To the best of knowledge, the present study was the first to explore the effect of DSE inoculation on the growth and rhizosphere-associated microbial composition of licorice seedlings under drought stress, in non-sterilized, sandy soil. In the present study, we hypothesize that DSE inoculation could either promote the growth of licorice plants or change the soil microbial composition of the licorice rhizosphere, and that DSE might have more positive effects under drought stress conditions than under control conditions. Therefore, we investigated the effects of DSE inoculation under drought resistance conditions on (1) plant growth, (2) plant photosynthesis, (3) antioxidant enzyme activities, (4) glycyrrhizic acid and glycyrrhizin accumulation, (5) Soil physicochemical properties, and (6) soil microbial composition. We expected our results to reveal the mechanism by which inoculated DSE could withstand the drought conditions that affected growth and medicinal ingredient accumulation, and their potential for improving the stress tolerance and symbiotic performance of plants during licorice cultivation, in drought-affected arid lands.

## Materials and Methods

### Biological Materials and Growth Medium

Three DSE fungi isolated from the roots of licorice, which grow naturally in arid farmland of North China, were used in this experiment. Three DSE species, namely *A. vagum*, *P. putaminum*, and *Fusarium acuminatum* were identified based on morphological characters and internal transcribed spacer (ITS) phylogeny; their ITS sequences are available at GenBank under the accession numbers MK392024 for *A. vagum*, MK601233 for *P. putaminum*, and MK583543 for *F. acuminatum*. These fungi were deposited in the culture collection of the Laboratory of Endangered Species Breeding Engineering, Institute of Medicinal Plant Development, Chinese Academy of Medical Sciences and Peking Union Medical College, Beijing, China. Seeds of licorice were provided by China National Traditional Chinese Medicine Corporation, and stored at 4°C.

The growth medium was a 1:2 (w:w) mixture of sand (<2 mm) and soil collected from the farmland of North China, in which licorice plants were naturally planted. The growth medium had an organic matter content of 21.57 mg/g, available nitrogen of 85.19 mg/kg, and available phosphorus of 7.90 mg/kg.

### Experimental Procedure

The experiment was conducted as a complete randomized factorial design with two factors. The first factor had four levels: non-inoculation control (CK), or inoculation with *A. vagum* (AV), *P. putaminum* (PP), or *F. acuminatum* (FA); and the second factor had two levels: well-watered (WW) and drought stress (DS). Each treatment consisted of five replicates with two plants per pot/replicate, thus totaling 40 experimental pots.

Licorice seeds were surface sterilized with 70% ethanol for 3 min and then treated with 2.5% sodium hypochlorite for 10 min, while providing agitation. The sterilized seeds were gently washed with sterile water several times, and aseptically planted onto water agar medium (containing 10 g/L agar) in Petri dishes, for germination at 27°C. Following pre-germination, the seedlings were transplanted to sterile plastic pots (13 cm diameter, 12 cm height, 2 seedlings for each pot) that were first filled with 800 g of non-sterile growth medium. Fungal inocula were prepared by aseptically growing DSE isolates in Petri dishes with potato dextrose agar (PDA) culture medium. For DSE inoculation, two 5 mm plugs excised from an edge of an actively growing colony on culture medium were inoculated at a 1 cm range close to the roots of licorice seedlings. Specifically, each experimental pot was first filled with 600 g soil, on which were two 5 mm DSE plugs, and then 200 g soil. All the inoculation processes were carried out on a clean bench, and all the experimental pots were kept in a growth chamber over a 14 h/10 h photoperiod, at a temperature of 27°C/22°C (day/night), and 60% mean relative humidity. Seedling growth occurred for a duration of 3 months.

One month later, half of the seedlings (both control and inoculation treatments) were subjected to WW treatment (70% field water capacity), and the other half were subjected to DS treatment (30% field water capacity). The drought stress treatments in this study were applied after taking the median value into account in the natural habitat of licorice plants in North China. The soil moisture was determined with a soil humidity recorder (L99-TWS-2, China). Water loss was daily supplemented with sterile distilled water to keep the desired field capacity by regular weighing.

### Photosynthetic Parameters

The day before the harvest of plants, the net photosynthesis rate (*Pn*), stomatal conductance (*Gs*), intercellular CO_2_ concentration (*Ci*), and respiration rate (*Rr*) for the third mature leaf from the top of the intact plants were measured, using a portable photosynthesis system (Li-6400X, Li-COR, Lincoln, United States). The chlorophyll concentration (*Chl*) of the third mature leaf from the top of the intact plants was measured using a SPAD-502 Chl meter (Konica Minolta Sensing, Osaka, Japan). This device measures the absorbance of leaves at a wavelength of 650 nm, at which the absorbance values for both *Chl* a and b could be maximal, while the absorbance was also measured at 940 nm. A “SAPD number” was calculated based on these two transmission values. Measurements were performed in a random order during the late morning (09:00–11:00 a.m.) period. Photosynthetic parameters for each replicate were the average of the two plants in each pot.

### Plant Growth Parameters

At the end of the growth period, plant heights and leaf numbers of each replicate with two plants in each pot were measured. Shoots and roots from each bottle were separately harvested, and roots were gently washed with tap water to remove sand. Individual root sections were allowed to float in water at a depth of approximately 1 cm in a plexiglass tray and scanned using a desktop scanner (EPSON Perfection V800 Photo, Japan). The total length, surface area, average diameter, and branch number were determined using the WinRHIZO image analysis system (Chen et al., 2012). Root and shoot biomass were determined by oven drying at 70°C for 48 h. The root and shoot biomass and root growth index were conducted as the sum of the two plants per pot. Soil that was strongly adhered to roots was defined as rhizosphere soil. Rhizosphere soil samples from each replicate were sieved using 2 mm sieves and divided into two subsamples: one subsample was dried at room temperature for soil physicochemical analyses, while the other subsample was frozen at −80°C for microbial community composition analysis.

### Antioxidant Enzyme Activity

Fresh leaf samples (0.5 g) from each plant were homogenized in 5 mL of 50 mM potassium phosphate buffer (pH 7.8), which contained chilled 0.2 mM EDTA and 2% (w/v) polyvinylpyrrolidone kept in an ice bath. A prechilled mortar and pestle were used for grinding. The homogenate was centrifuged at 15000 rpm for 30 min and the supernatant was used for enzyme assays. Activities of superoxide dismutase (SOD) activity was analyzed via nitroblue tetrazolium photochemical reduction, and the catalase (CAT) activity was determined by measuring the consumption of hydrogen peroxide at a wavelength of 240 nm, as a unit of enzyme activity. The activity of peroxidase (POD) determined using the using guaiacol method (Harrach et al., 2008).

The malondialdehyde (MDA) concentration was determined using the thiobarbituric acid (TBA) method described by Peever and Higgins (1989). Briefly, 0.5 g fresh leaf samples were homogenized in 10% trichloroacetic acid (TCA) (5 mL), and centrifuged at 12000 × *g* for 10 min. A mixture containing the supernatant (2 mL) and 0.5% thiobarbituric acid (TBA) (2 mL) was placed in a boiling water bath. After 15 min, the mixture was rapidly cooled and then centrifuged at 12000 × *g* for 10 min. The absorbance of the supernatant was determined at 450, 532, and 600 nm using a spectrometer. The following formula was used to calculate the MDA concentration:

C(μmol/L)=6.45(OD-532OD)60-0.56OD450

Soluble protein content was measured using Coomassie brilliant blue G-250 reagent, described by Zhang et al. (2015). Briefly, 0.5 g fresh leaf samples were ground into the homogenate using quartz sand in 5 mL of 50 mM ice-cold phosphate buffer (pH 7.0) at 4°C, in a precooled mortar. The homogenate was centrifuged at 4,000 × *g* for 10 min at 4°C, after which the supernatant was collected. The absorbance of the reaction mixture was measured at 595 nm using a spectrophotometer (752 N model, Shanghai INESA Instrument Analytical Instruments Co., Ltd., China). Bovine serum albumin (BSA) was used as a standard.

### Active Ingredients in the Roots

Dried root samples after uniformly mixing two plants per pot were ground into powder using a mortar and pestle, and passed through a 40-mesh sieve. About 0.05 g of sample was weighed and extracted in 10 mL methanol/water (70:30) for 30 min in an ultrasonic bath at 25°C. The extract solution was cooled to a temperature of 25°C and filtered using a 0.45 μm filter. A 10–μL aliquot of the filtrate was subjected to separation via high–performance liquid chromatography, using a reverse phase C_18_ symmetry column (4.6 mm × 250 mm, pore size 5 μm; Waters Corp., Milford, MA, United States). The mobile phase comprised a gradient of deionized water:phosphoric acid (100:0.05, v/v) and acetonitrile. The separation process was conducted in the gradient elution mode (Supplementary Table S1) at 25°C, at a flow rate of 1.0 mL/min. The eluted compounds were detected spectrophotometrically at 237 nm, using a 2998 PDA photodiode array detector. Glycyrrhizic acid and glycyrrhizin were purchased from China National Institutes for Food and Drug Control. Their stock solutions were diluted with 70% aqueous methanol to obtain solutions with an appropriate concentration for calibration purpose (Zhang et al., 2013).

### DSE Root Colonization

Fresh roots were washed in tap water and cut into 0.5-cm long segments. For each sample, 10 randomly selected root segments were examined to confirm that the roots were colonized by respective fungal inocula. The root segments were surface sterilized by dipping in 70% ethanol for 5 min and then 5% sodium hypochlorite for 5 min and then washing in sterile distilled water. These roots were then transferred to PDA culture medium and kept at 27°C in the dark (Li et al., 2018). In addition, the segments were cleared in 10% (w/v) potassium hydroxide and stained with 0.5% (w/v) acid fuchsin (Phillips and Hayman, 1970). Assessment of fungal colonization was conducted on each sample using the glass slide method, in which 20 randomly selected 0.5-cm long root segment units were examined microscopically at 20x and 40x magnification (Biermann and Linderman, 1981). DSE colonization rate (%) was calculated according to the following formula:

Colonization rate (%)=(length of colonization root segments/total length of root segments)×100%.

### Composition of Soil Microbial Community

The composition of soil microbial community in the rhizosphere was determined by analyzing the composition of ester-linked phospholipid fatty acids (PLFAs) in soils. Briefly, lipids from roughly weighed (8.0 g) frozen soil subsamples were extracted overnight using the modified Bossio and Scow (1998) method, using 23 mL of chloroform:methanol:phosphate buffer (1:2:0.8 v/v/v) solution. The chloroform was transferred to a silica gel column (0.5 g silicic acid, 3 mL; HF BOND ELUT e SI, Varian, Inc., Darmstadt, Germany), and lipids were sequentially eluted with 5 mL chloroform (NLFAs), 20 mL acetone (glycolipids), and 5 mL methanol (PLFAs). Methanol solution was collected, and the gas was allowed to get emitted in the presence of nitrogen. The phospholipids were sequentially saponified and methylated, forming fatty acid methyl esters (FAMEs). Individual FAMEs were identified and quantified using a gas chromatograph (GC, America, Agilent 6890N), using MIDI software (MIDI Inc., Newark, DE, United States) and the software package Sherlock MIS Version 4.5 (MIDI Inc., Newark, DE, United States). The MIDI software automatically controlled all gas chromatographic operations, including calibration, subsequent sample sequencing, peak integration, and naming. Calibration standards contained a mixture of straight chain saturated and hydroxy FAMEs with 10–20 carbon atoms (MIDI Part No. 1208).

The composition of soil microbial community was determined by using the following PLFAs: 14:1 iso w7c, 14:0 iso, 14:0 anteiso, 15:1 iso w9c, and 15:1 iso w6c for gram- positive (G+) bacteria, and 14:1 w9c, 14:1 w8c, 14:1 w7c, 14:1 w5c, 15:1 w9c, 15:1 w8c, 15:1 w 7c, and 15:1 w6c for gram-negative (G–) bacteria, whereas 16:0 10-methyl, 17:1 w7c 10-methyl, 17:0 10-methyl, and 18:1 w7c 10-methyl for actinomycetes. The fatty acids 18:2 w6c and 18:1 w9c were summed to indicate the fungi. The fatty acid 16:1 w5c was assigned as marker for AM fungi.

### Soil Physicochemical Properties

A dried soil sample (0.2 g) was digested in 10 mL of a mixture containing perchloric acid (12.7 mol/L), sulfuric acid (18 mol/L), and water in the ratio of 10:1:2 using the Mars 6 microwave reaction system (CEM Corporation, Matthews, NC, United States) until a clear liquid was obtained. The content of soil organic matter, available nitrogen (N), available phosphorus (P), and available potassium (K) content was quantified via oxidization with dichromate in the presence of sulfuric acid (Rowell, 1994), and by using the alkaline hydrolysis-diffusion, chlorostannous-reduced molybdophosphoric blue method (Olsen et al., 1954), and flame photometer (Jackson, 1973) method.

### Statistical Analysis

A two-way analysis of variance was used to analyze the effects of DSE, water, and their interaction on plant growth parameters, photosynthetic parameters, antioxidant parameters, glycyrrhizic acid and glycyrrhizin contents, and microbial community composition, and mineral nutrient contents in rhizosphere soil. The values reported in figures are means of at least three replicates. The differences between the means among different treatments were compared using Duncan’s multiple-range tests at *p* < 0.05. Variation partitioning was performed to estimate the size of effect that each factor has on plant growth and active ingredients. SPSS 21.0, Canoco 4.5, RStudio packages vegan (Borcard et al., 2011), and Kaleida Graph 4.5 were used for statistical analyses and plotting.

## Results

### DSE Colonization Observation

After harvesting, DSE hyphal and microsclerotial structures were observed in all the tested root samples of licorice plants under all treatments. Total root colonization was 16.6% in control plants, 43.3% in AV-inoculated plants, 36.6% in PP-inoculated plants, and 16.6% in FA-inoculated plants under WW conditions, respectively. However, total root colonization was 3.3% in control plants, 43.3% in AV-inoculated plants, 20.0% in PP-inoculated plants, and 23.3% in FA-inoculated plants under DS conditions, respectively (Supplementary Figures S1, S2).

### Plant Biomass Production

**FIGURE 1:**
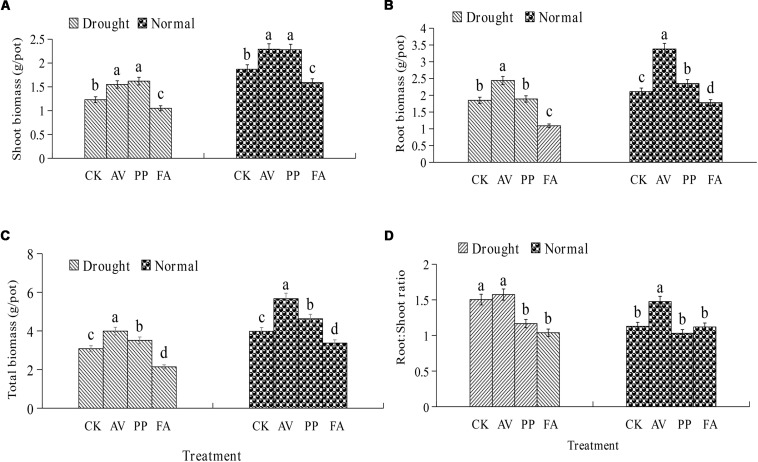
Effects of dark septate endophyte (DSE) inoculation and water treatment on the plant biomass of licorice plants. The error bars represent the standard error (SE). Different letters above the error bars indicate significant difference at *P* < 0.05 by Duncan’s multiple-range tests. CK indicates non-inoculated plants. AV, *A. vagum*. PP, *P. putaminum*. FA, *F. acuminatum*. The shoot biomass of licorice plants **(A)**, root biomass of licorice plants **(B)**, total biomass of licorice plants **(C)**, root:shoot ratio of licorice plants **(D)**.

The inoculation of DSE fungi had significant effects on the total, root, and shoot biomass levels, and the root:shoot ratio, under different water-related conditions (Figure 1). AV inoculation resulted in a significant increase in the total biomass, root biomass, and shoot biomass levels, regardless of the water regime, but the root:shoot ratio was increased only under WW conditions. PP inoculation increased the total, root, and shoot biomass levels, while FA inoculation decreased the total, root, and shoot biomass levels under WW conditions. In the presence of DS, the inoculation of PP increased the shoot and total biomass levels, and decreased the root:shoot ratio; however, FA inoculation decreased the total, root, and shoot biomass levels, and the root:shoot ratio, as compared to the values for control plants (Figure 1). No significant interactions were observed between DSE and water in plant biomass (Table 1).

**TABLE 1:** Two-way analysis of variance for the effects of dark septate endophyte (DSE) and water treatment on the growth and physiological parameters of licorice plants.

	**DSE**		**Water**		**DSE × Water**	
	***F***	***P***	***F***	***P***	***F***	***P***
Total biomass (g/pot)	25.32	**<0.001**	50.84	**<0.001**	0.88	0.462
Root length (cm)	165.68	**<0.001**	232.11	**<0.001**	9.043	**<0.001**
Root branch (No.)	529.99	**<0.001**	273.93	**<0.001**	137.21	**<0.001**
Glycyrrhizic acid (%)	22.32	**<0.001**	7.28	**0.003**	56.16	**<0.001**
Glycyrrizin (%)	1.448	0.254	2.40	0.134	1.05	0.391
SPAD value	3.85	**0.021**	0.58	0.450	0.464	0.634
Pn (μmol/m^2^/s)	2.34	0.098	94.07	**<0.001**	7.71	**0.001**
Gs (mol/m^2^/s)	1.54	0.230	18.58	**<0.001**	3.03	**0.049**
Ci (μmol/mol)	2.54	0.081	11.87	**<0.001**	2.39	0.094
Rr (μl)/(h ⋅ g)	29.07	**<0.001**	32.61	**<0.001**	5.56	**0.005**
SOD (U/g ⋅ FW ⋅ h)	6.66	**0.011**	0.046	0.833	2.49	0.084
CAT (U/g ⋅ FW ⋅ h)	24.69	**<0.001**	2.80	0.107	3.92	**0.021**
MDA (μmol/gFW)	2.62	0.064	4.25	**0.012**	2.33	0.084
Soluble protein	2.71	0.058	2.22	0.098	4.53	**0.022**
(mg/gFW)						
POD (U/g ⋅ FW ⋅ h)	5.62	**0.035**	1.96	0.092	2.25	0.077

*Significant *P*-values are in bold.*

### Plant Growth Parameters

**FIGURE 2:**
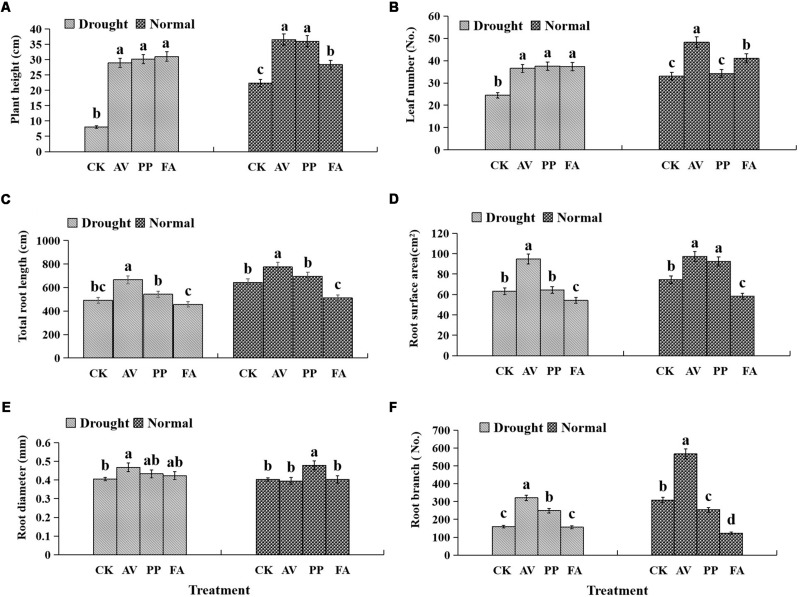
Effects of dark septate endophyte (DSE) inoculation and water treatment on the plant growth parameters of licorice plants. The error bars represent the standard error (SE). Different letters above the error bars indicate significant difference at *P* < 0.05 by Duncan’s multiple-range tests. CK indicates non-inoculated plants. AV, *A. vagum*. PP, *P. putaminum*. FA, *F. acuminatum*. The heights of licorice plants **(A)**, leaf numbers of licorice plants **(B)**, total root length of licorice plants **(C)**, root surface area of licorice plants **(D)**, root diameter of licorice plants **(E)**, root branch numbers of licorice plants **(F)**.

DSE inoculation significantly increased the plant height, regardless of the watering regime, as compared to that of the control plants (Figure 2A), and significantly increased the leaf number under DS conditions. However, only AV and FA inoculation caused a significant increase in the leaf number under WW conditions (Figure 2B).

The significant interactions between DSE and water that affected the total root length and root branch numbers were found (Table 1). Under WW conditions, AV inoculation increased the total root length, surface area, and root branch number; PP inoculation increased the root surface area and diameter, and decreased the root branch number; however, FA decreased the total root length, surface area, and branch number, as compared to the values for the control plants (Figures 2C–F). Under DS conditions, AV inoculation increased the total root length, surface area, diameter, and branch number; however, PP inoculation only increased the root branch number, and FA inoculation only decreased the root surface area, as compared to the values for the control plants (Figures 2C–F).

### Photosynthetic Parameters in Leaves

**FIGURE 3:**
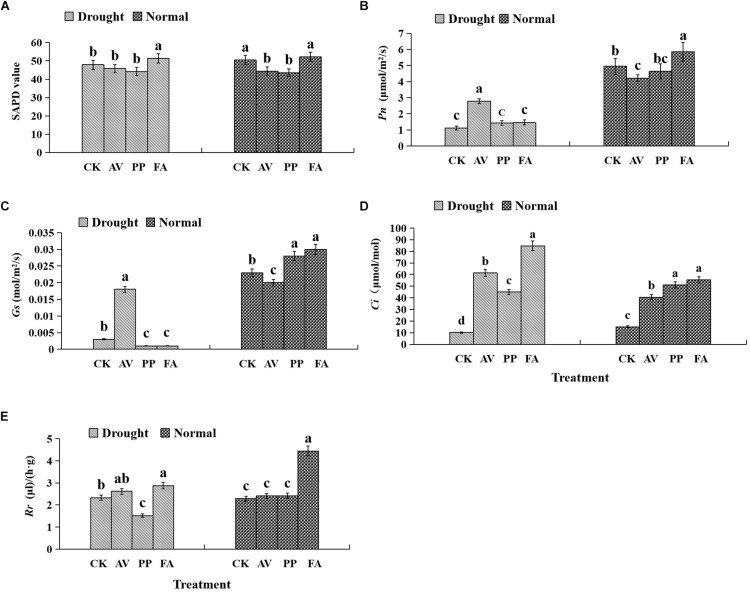
Effects of dark septate endophyte (DSE) inoculation and water treatment on the photosynthetic parameters in licorice leaves. The error bars represent the standard error (SE). Different letters above the error bars indicate significant difference at *P* < 0.05 by Duncan’s multiple-range tests. CK indicates non-inoculated plants. AV, *A. vagum*. PP, *P. putaminum*. FA, *F. acuminatum*. SPAD, chlorophyll content. *Pn*, net photosynthesis rate. *Gs*, stomatal conductance. *Ci*, intercellular CO_2_ concentration. *Rr*, respiration rate. The SPAD of licorice plants **(A)**, *Pn* of licorice plants **(B)**, *Gs* of licorice plants **(C)**, *Ci* of licorice plants **(D)**, *Rr* of licorice plants **(E)**.

The photosynthetic parameters in leaves were affected significantly by DSE inoculation regardless of the water regime (Table 1). Inoculation with AV and PP decreased SPAD values, while FA inoculation had no significant effects on SPAD values under WW conditions, as compared to those in control plants (Figure 3). However, only FA inoculation increased SPAD values under DS conditions, as compared to those of control plants. Inoculation with all the tested DSE increased *Ci* values, as compared to those of control plants (Figure 3). The interaction of DSE and water significantly affected the expression of *Pn*, *Gs*, and *Rr* in leaves (Table 1). Under WW conditions, AV inoculation decreased the expression of *Pn* and *Gs*; PP alone increased the expression of *Gs*, whereas FA increased the expression of *Pn*, *Gs*, and *Rr*, as compared to that of control plants (Figure 3). Under DS conditions, AV inoculation increased *Pn* and *Gs* expression, and PP decreased *Gs* and *Rr* expression, but FA alone increased *Rr* expression, as compared to that of control plants (Figure 3).

### Antioxidant Enzyme Activities and Osmotic Materials in Leaves

**FIGURE 4:**
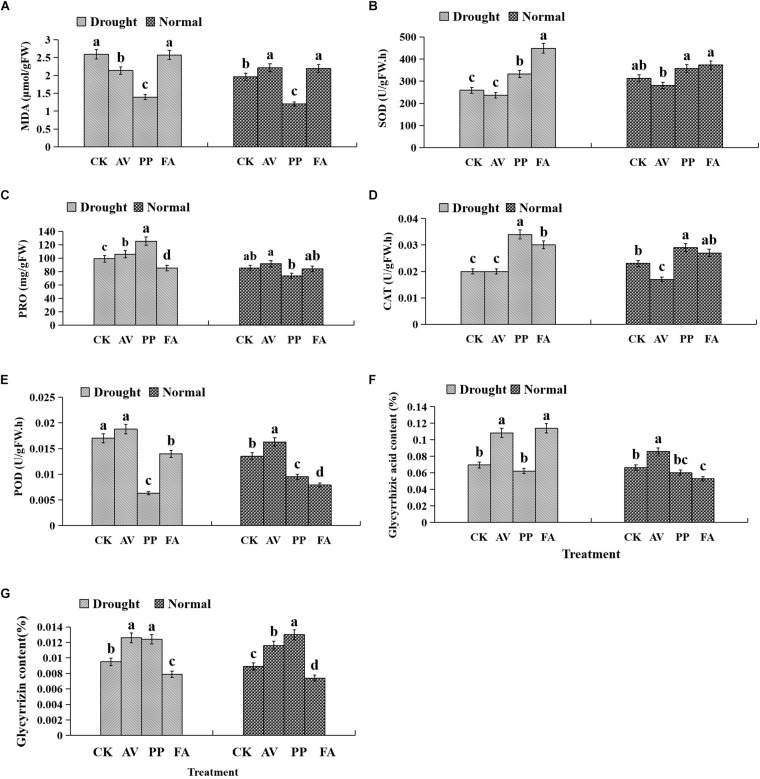
Effects of dark septate endophyte (DSE) inoculation and water treatment on the antioxidant parameters in leaves and active ingredient in roots of licorice plants. The error bars represent the standard error (SE). Different letters above the error bars indicate significant difference at *P* < 0.05 by Duncan’s multiple-range tests. CK indicates non-inoculated plants. AV, *A. vagum*. PP, *P. putaminum*. FA, *F. acuminatum*. The MDA content of licorice plants **(A)**, SOD activity of licorice plants **(B)**, soluble protein (PRO) content of licorice plants **(C)**, CAT activity of licorice plants **(D)**, POD activity of licorice plants **(E)**, glycyrrhizic acid content of licorice plants **(F)**, glycyrrhizin content of licorice plants **(G)**.

Inoculation with FA significantly increased the MDA content, whereas inoculation with PP decreased MDA content, regardless of the water regime. Under WW conditions, AV inoculation increased the MDA content and POD activity, and decreased CAT activity; PP increased the activity of CAT and decreased the activity of POD. However, the use of FA alone decreased POD activity, as compared to that of control plants (Figure 4). Under DS conditions, the inoculation of AV alone increased soluble protein content, and that of PP alone increased soluble protein content, and SOD and CAT activities, whereas FA increased SOD and CAT activities, and decreased the soluble protein content and POD activity, as compared to that of control plants (Figure 4). Significant interactions between DSE and water affected the CAT activity and soluble protein content (Table 1).

### Active Ingredient Contents

The glycyrrhizic acid content of roots was affected significantly by DSE inoculation, regardless of the water regime (Table 1). Under WW conditions, inoculation with AV increased the glycyrrhizic acid and glycyrrhizin content, while that with PP only decreased the glycyrrhizin content; however, FA decreased the glycyrrhizic acid and glycyrrhizin content, as compared to that of control plants (Figures 4F–G). Under DS conditions, the glycyrrhizic and glycyrrhizin content was increased by AV inoculation, while only glycyrrhizin content was increased by PP inoculation; however, inoculation with FA increased glycyrrhizic acid levels and decreased glycyrrhizin levels, as compared to those of control plants (Figures 4F–G). The interactions between DSE and water significantly affected the glycyrrhizic acid content (Table 1).

### Soil Microbial Community Composition

**FIGURE 5:**
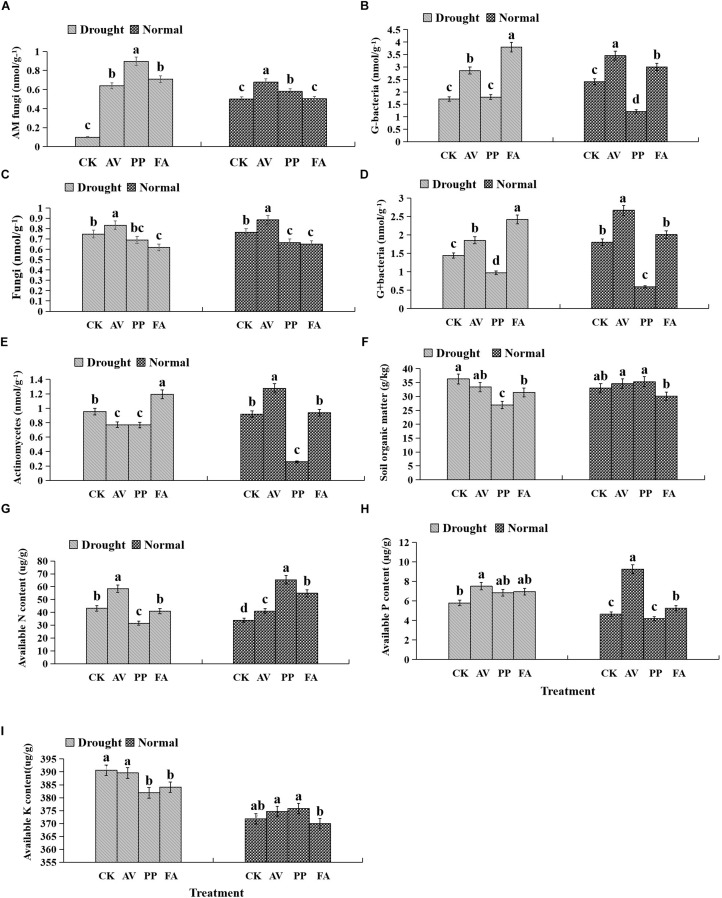
Effects of dark septate endophyte (DSE) inoculation and water treatment on soil physicochemical properties and microbial community composition in rhizosphere soil. The error bars represent the standard error (SE). Different letters above the error bars indicate significant difference at *P* < 0.05 by Duncan’s multiple-range tests. CK indicates non-inoculated plants. AV, *A. vagum*. PP, *P. putaminum*. FA, *F. acuminatum*. SOM = soil organic matter. SAP = soil available P. SAN = soil available N. SAK = soil available K. AM = AM fungi. Fungi = other fungi. G– = Gram-negative bacteria. G+ = Gram-positive bacteria. ACT = Actinomycetes. AM fungi of rhizosphere soil **(A)**, G– of rhizosphere soil **(B)**, fungi of rhizosphere soil **(C)**, G+ of rhizosphere soil **(D)**, actinomycetes of rhizosphere soil **(E)**, soil organic matter of rhizosphere soil **(F)**, soil available N of rhizosphere soil **(G)**, soil available P of rhizosphere soil **(H)**, soil available K of rhizosphere soil **(I)**.

AM fungi and G− bacterial levels were affected significantly by DSE inoculation, regardless of the water regime (Table 2). Under WW conditions, inoculation with AV increased the number of G+ bacteria, G− bacteria, fungi, AM fungi, and actinomycetes; inoculation with PP decreased the number of G+ bacteria, G− bacteria, fungi, and actinomycetes, and increased the AM fungal levels. However, inoculation with FA only increased the number of G− bacteria and decreased that of fungi, as compared to that of control plants (Figures 5A–E). Under DS conditions, inoculation with AV increased the number of G+ bacteria, G− bacteria, AM fungi, and fungi, and decreased that of Actinomycetes; PP inoculation only increased the number of AM fungi and G− bacteria, and decreased the number of G+ bacteria, whereas FA inoculation increased the number of AM fungi, G+ bacteria, G− bacteria, and actinomycetes, and decreased the number of fungi, as compared to that of control plants (Figures 5A–E). The interactions between DSE and water significantly affected the number of AM fungi (Table 2).

**TABLE 2:** Two-way analysis of variance for the effects of dark septate endophyte (DSE) and water treatment on microbial composition and physicochemical properties in rhizosphere soil of licorice plants.

	**DSE**		**Water**		**DSE × Water**	
	***F***	***P***	***F***	***P***	***F***	***P***
AM fungi (nmol/g^–1^)	6.482	**0**.**004**	43.108	**<0.001**	374.523	**<0.001**
Fungi (nmol/g^–1^)	2.341	0.067	1.432	0.294	1.547	0.235
G− bacteria (nmol/g^–1^)	5.957	**0**.**006**	0.231	0.817	0.866	0.423
G+ bacteria (nmol/g^–1^)	0.581	0.527	0.955	0.339	1.085	0.364
Actinomycetes (nmol/g^–1^)	1.857	0.186	1.233	0.321	2.074	0.080
Organic matter (g/kg)	4.925	**0**.**044**	0.200	0.174	6.134	**0.019**
Alkaline N (μg/g)	0.133	0.720	0.126	0.943	0.654	0.592
Available P (μg/g)	7.752	**0**.**005**	1.757	0.197	0.771	0.572
Available K (μg/g)	3.184	0.091	5.893	**0.038**	0.127	0.943

*Significant *P*-values are in bold.*

### Soil Physicochemical Properties

Inoculation with DSE significantly affected the level of soil organic matter and available P, regardless of the water regime (Table 2). Under WW conditions, inoculation with AV increased the soil available N and available P, while inoculation with PP only increased the soil available N; however, inoculation with FA alone increased soil available N and P, and decreased the soil organic matter content, as compared to that for control plants (Figures 5F–I). Under DS conditions, inoculation with AV increased soil available N and available P, and inoculation with PP decreased the organic matter and available N in the soil; however, inoculation with FA alone decreased the amount of soil organic matter, as compared to that for control plants (Figures 5F–I). The interactions between DSE and water significantly affected the soil organic matter content in the soil (Table 2).

### Variation Partitioning of Growth Parameter and Active Ingredient Contents

**FIGURE 6:**
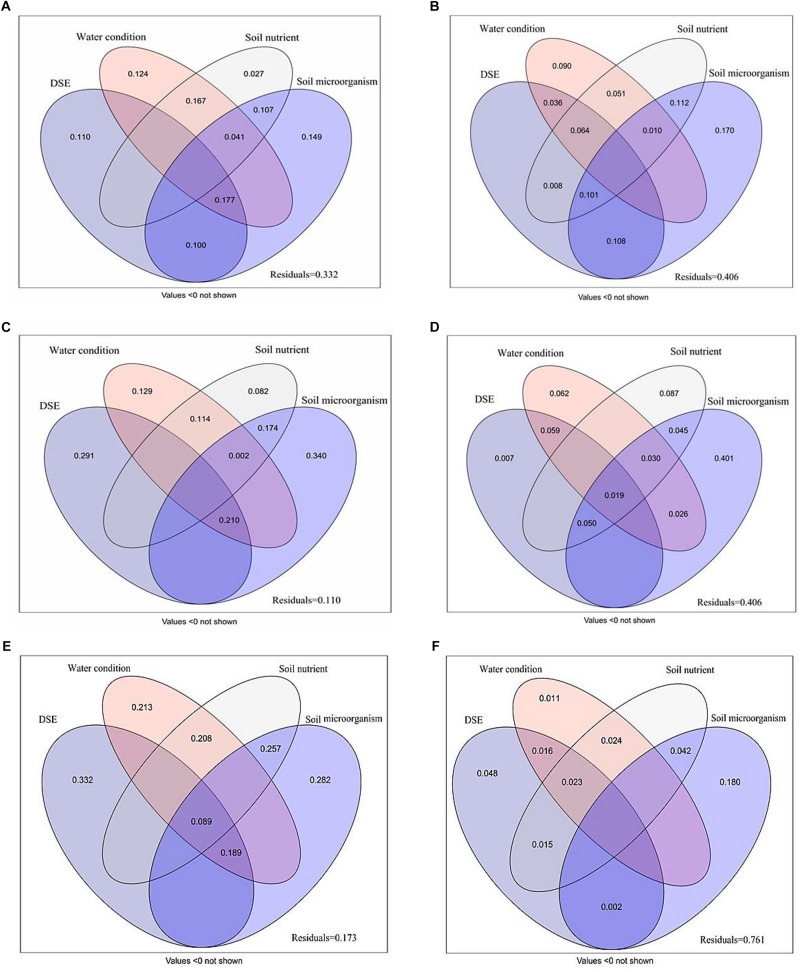
Variation partitioning of DSE species, water condition, soil nutrient properties and microorganism on plant biomass **(A)**, shoot morphology **(B)**, root morphology **(C)**, active ingredient contents **(D)**, photosynthetic parameters **(E)**, and antioxidant parameters **(F)**. DSE, DSE species; Soil nutrient, nutrient content (including soil organic matter, available N, available P and available K); Soil microorganism (including AM fungi, Fungi, G–, G+ and Actinomycetes). Values below 0 are not shown.

Variance partitioning analysis was conducted to quantify the association between DSE, water, soil nutrient properties and microorganism to plant biomass, growth parameters, photosynthetic parameters, antioxidant parameters, and active ingredient (Figure 6). A mix of DSE, water, soil nutrient properties and microorganism caused a diversification of 66.8% in the plant biomass; of this, soil microorganism and DSE were attributable for 14.9 and 11.0% of the diversification, respectively (Figure 6A). A mix of DSE, water, soil nutrient properties, and microorganism caused 59.4% diversification in the shoot morphology; soil microorganism might be a key factor, as it accounts for 17.0% of the observed diversification (Figure 6B). The main variations in root morphology are attributable purely to the effects of DSE and soil microorganism, which accounted for 29.1 and 34.0% of the variations, respectively, whereas a mix of DSE, water, soil nutrient properties, and microorganism caused 89.0% of the variations observed in root morphology (Figure 6C). Soil microorganism accounted for 40.1% of the diversification in active ingredient content, and represent the most influential factor, whereas a mix of DSE, water, soil nutrient properties, and microorganism caused 59.4% of diversification (Figure 6D). A mix of DSE, water, soil nutrient properties, and microorganism caused a diversification of 82.7% in the plant photosynthetic parameters; of this, DSE and soil microorganism were attributable for 33.2 and 28.2% of the diversification, respectively (Figure 6E). A mix of DSE, water, soil nutrient properties, and microorganism caused 23.9% diversification in the antioxidant parameters; soil microorganism might be a key factor, as it accounts for 18.0% of the observed diversification (Figure 6F).

## Discussion

Although some studies have reported that DSE can act as plant growth promoters (Zhu et al., 2015; He et al., 2019; Xie et al., 2019), their effects on plants are still limited under drought stress conditions (Li et al., 2018). The results of existing studies on the effects of inoculation of DSE on plant growth under water deficiency conditions are variable. Inoculation of DSE caused negative to neutral and positive effects on plant growth under water deficiency conditions (Perez-Naranjo, 2009; Santos et al., 2017; Zhang et al., 2017). In the present study, typical DSE hyphae and microsclerotia were observed in root samples after all the treatments were performed, which indicates that the three DSE species are effective root colonizers, even under drought conditions. In addition, the inoculation of AV and PP caused significant positive effects on plant biomass, whereas that of FA had a negative effect on plant biomass, regardless of water conditions. Our results also showed that DSE had significant and direct effects on plant biomass, whereas the combination of DSE and water did not significantly influence plant biomass. This is consistent with the findings of previous studies, which showed that the interactions of DSE with plants might be affected by the DSE species (Mandyam and Jumpponen, 2005; Li X. et al., 2019).

In the present study, the effects of DSE on root morphological traits depend on the DSE species and water conditions. Specifically, AV exhibited significant positive effects on root morphological traits regardless of the water regime, and PP exhibited significant positive effects on root branch numbers under drought stress; whereas FA had a significant negative effect on the root surface area regardless of the water regime. Our results indicated that the combination of DSE and water significantly positively influenced the total root length and root branch number. Our results also indicated that DSE inoculation improved the performance of host plants by affecting their root morphology under drought stress (González-Teuber et al., 2018; Li et al., 2018). Moreover, inoculation with DSE had significant positive effects on plant heights and leaf numbers under drought stress. Although DSE only colonized in plant roots, various groups of microbes interacted with the leaves and stems of the plant (Harman, 2011; Vergara et al., 2017).

Our current knowledge suggests that plant growth and development is correlated with photosynthetic capacity. Our results showed that drought stress decreased the *Pn*, *Gs*, *Ci*, and *Rr* levels, though their levels were partly recovered upon DSE inoculation. Specifically, AV significantly increased *Pn*, *Gs*, and *Ci* levels, whereas PP and FA significantly increased *Ci* levels. These results suggested that the inoculation of DSE might affect the open status of stomata and intercellular concentration of CO_2_ in leaves under drought stress conditions. In addition, although drought stress did not significantly affect the SPAD values, FA significantly increased SPAD values under drought stress conditions in the present study. Hosseini et al. (2017) reported that the levels of plant adaptability, root length, root volume, and leaf chlorophyll content were significantly increased by *Piriformospora indica*, owing to the higher number of water and nutrient absorption sites. Ahmadvand and Hajinia (2018) also indicated that the inoculation of *P. indica* enhanced chlorophyll contents by 27.18% in millet plant under severe water stress, as compared to that in control plants. In addition, glycyrrhizic acid and glycyrrhizin were determined to be the main active ingredients in licorice roots in this study. Our results revealed that the use of DSE alone, and a combination of DSE and water had significant direct effects on glycyrrhizic acid levels. Specifically, AV and FA increased glycyrrhizic acid content, whereas AV and PP increased glycyrrhizin content under drought stress. These findings suggested that the presence of endophytic fungi reduces the adverse effects of individual and combined stresses on plant growth and active ingredients accumulation (Xie et al., 2018; Li X. et al., 2019).

In the present study, our results showed that DSE had significantly and directly affected SOD and CAT levels, and the combination of DSE and water significantly positively affected the soluble protein and SOD levels. SOD, CAT, and POD are reportedly the most important enzymes involved in the removal of reactive oxygen species (ROS) (Hosseini et al., 2018). The increased activities of SOD and CAT in the PP-inoculated and FA-inoculated plants, and the increase of soluble protein content in the AV-inoculated and PP-inoculated plants at water stress indicated that the antioxidant enzyme activity and soluble protein level were enhanced to remove ROS under drought stress conditions (Hosseini et al., 2018; Wang et al., 2019). Using soluble proteins as compatible osmolytes can facilitate osmotic adjustment, leading to an increased tolerance to dehydration (Doganlar et al., 2010). Soluble protein accumulation in AV-inoculated and PP-inoculated plants reduced the negative influences of drought stress by counterpoising the solute potential, which then contributes to cell growth. Similar results were also reported regarding response of tomato seedlings to DSE inoculation under Zn and Cd stress (Zhu et al., 2018). Moreover, Zhu et al. (2018) found that DSE significantly enhanced the POD activity under metal stress conditions, however, PP and FA inoculation significantly decreased the POD activity in our study. This might be attributable to the fact that the POD activity depends upon the DSE strains and growth conditions. MDA, which acts as a biomarker of oxidative stress, can be used to assess the extent of damage caused by oxidative stress (Khan et al., 2012). The decline in MDA levels in AV-inoculated and PP-inoculated plants under drought stress conditions clearly implies that the DSE-inoculated plants protection against the detrimental effects of drought (Xu et al., 2017).

In contrast to the vast information regarding AM fungi, little is known regarding the effect and mechanism of DSE on soil nutrient properties and microbial community composition. In the present study, DSE had significantly positive, direct effects on available N and available P in the soil, and had direct negative effects on organic matter in the soil. The interactions between DSE and water also positively influenced soil organic matter and available P content. A similar response was also observed for licorice seedlings grown in sterilized soil after DSE inoculation (He et al., 2019). Two possible reasons can explain the effects of inoculating DSE in soil nutrient properties. First, DSE inoculation improved the root system and N and P absorption by plants, which consequently led to the depletion of these common nutrients in the soil (Vergara et al., 2017; Li X. et al., 2019). Second, DSE could act as decomposers and convert soil organic nutrients into available forms to promote the growth and tolerance of plants to stressful conditions (Surono and Narisawa, 2017; He et al., 2019). As a bridge linking the plant and soil environment, DSE organisms increase the interactions between plants and soil, and expands the available N and P, because they secrete several enzymes for the mineralization of organic N and insoluble P in the soil into available forms; thus, they promote the growth and tolerance of plants (Berthelot et al., 2016; Vergara et al., 2017; He et al., 2019).

Soil moisture has significant effects on the soil microbiota, however, its effects are varied depending on the microbiota (Cavagnaro, 2016). In our study, AV significantly increased the amount of soil fungi and G+ bacteria contents, and PP decreased soil G+ bacteria contents, whereas FA increased the amount of soil G+ bacteria contents, as compared to that observed with control plants under drought stress. Our results also showed that DSE had direct and significant effects on the soil AM fungi and G− bacterial content, and the combination of DSE associated with water significantly positively influenced soil AM fungi. Numerous studies have confirmed that AM fungi can improve plant growth by alleviating the stress caused by water stress (Barea et al., 2011), and increase nutrient supply and plant growth under drought conditions (Smith and Smith, 2012; Bitterlich et al., 2018). Carrasco et al. (2010) indicated that G− bacteria were more predominant than G+ bacteria in heavy metal-contaminated soils, because the former showed a greater adaptability to adverse conditions; the predominance of G+ bacteria could have a positive effect on the proliferation of AM fungi (Artursson et al., 2006). In addition, AV and PP significantly decreased the number of actinomycetes, while FA increased the number of actinomycetes as compared to that in control plants under drought stress conditions in our study. Actinomycetes, an important group of G+ bacteria, are one of the major components of rhizosphere microbial populations, and are useful for soil nutrient cycling, as well as plant growth promotion (Sreevidya et al., 2016). Franco-Correaa et al. (2010) observed that the inoculation of AM fungi improved the early establishment of actinomycetes strain *Streptomyces* MCR9 in the clover rhizosphere, and *Streptomyces* MCR9 and *Streptomyces* MCR26 stimulated the germination and mycelial development of AM fungi spores, while *Thermobifida* MCR24 inhibited spore germination but stimulated mycelial development. Further, our results also showed that available N had significant, direct effects on soil fungi, and available P had significant, direct effects on soil fungi and G− bacteria, while soil organic matter significantly positively affected the soil AM fungi and G− bacterial levels. Haas et al. (2018) suggested that soil microbial composition could be significantly influenced by nutrient inputs. The rhizosphere microbial community composition is reportedly dependent on the soil nutrient status (Fierer et al., 2009), and the quantity and quality of root exudates (Marschner and Timonen, 2005; Xie et al., 2019), which in turn are influenced by the nutritional and physiological status of the plant (Neumann and Römheld, 2007; Li X.G. et al., 2019). Previous studies have indicated that drier soils are more enriched in G− bacteria and fungi, and that wetter soil is more enriched in G+ bacteria (Drenovsky et al., 2010; Ma et al., 2015; Hinojosa et al., 2019). Based on the results of variance partitioning analysis, 17.0, 34.0, 14.9, 40.1, 28.2, and 18.0% variations in shoot morphology, root morphology, plant biomass, active ingredient, photosynthetic parameters, and antioxidant parameters, respectively, were attributable to the presence of certain soil microorganisms. These findings suggest the possibility that the constituents of altered soil microbiota might contribute to plant growth and survival under drought conditions (Santos-Medellín et al., 2017). To our knowledge, the reasons for the increase in the growth and drought resistance of licorice plants might be attributable to the improvement in plant physiological characteristics and root structure profiles following root colonization with the DSE. These changes could be related to the modification in mineral nutrients in the rhizospheric soil and the composition of the microbial community after DSE inoculation. In addition, based on the results of variance partitioning analysis, 33.2, 40.6, 11.0, 40.6, 17.3, and 76.1% of variations were still observed in the plant biomass, shoot index, root index, active ingredient content, photosynthetic parameters, and antioxidant parameters, which indicates that certain unexplored factors might also significantly affect the growth and the accumulation of the active ingredients of licorice plants, such as the DSE inoculation volume and interactions of DSE with other microbes.

## Conclusion

In this study, we first assessed the effects of DSE on the performance of licorice grown in non-sterile soil under water deficit stress. Although drought stress decreased the growth of licorice plants, the decrease in growth was partly recovered by DSE inoculation. Specifically, the inoculation of *A. vagum* and *P. putaminum* significantly increased plant biomass under drought stress. Meanwhile, *A. vagum* increased the glycyrrhizic acid and glycyrrhizin content, whereas *P. putaminum* increased the glycyrrhizin content. Although *F. acuminatum* significantly decreased the plant biomass and glycyrrhizin levels, this fungus increased glycyrrhizic acid levels under water deficit stress. Interestingly, DSE inoculation significantly influenced the composition of soil mineral nutrients and microbial communities; specifically, all tested DSE species significantly increased the AM fungal content, while AV and FA significantly increased the number of G− bacteria under water deficit conditions. Licorice can be used as an important herbal medicine or plant for the reclamation of drought-affected soils; microbes living in the rhizosphere of licorice can form a mutualistic association and coordinate their involvement in plant adaptations to stress tolerance. As *A. vagum* exhibited positive effects on plant biomass, growth, physiological parameters, and active ingredient content in licorice plants under drought stress conditions, it was considered to be the most effective fungus for the cultivation of licorice plants in drylands.

## Data Availability Statement

All datasets generated for this study are included in the manuscript/Supplementary Files.

## Author Contributions

CH and WW conceived and designed the experiments and wrote the manuscript. CH performed the experiments. CH and JH analyzed the data.

## Conflict of Interest

The authors declare that the research was conducted in the absence of any commercial or financial relationships that could be construed as a potential conflict of interest.
