# Supplementary material for: Plant Growth and Soil Microbial Impacts of Enhancing Licorice With Inoculating Dark Septate Endophytes Under Drought Stress
Source: Front Microbiol. 2019 Oct 9;10:2277. doi: 10.3389/fmicb.2019.02277 (PMC6794389; doi:10.3389/fmicb.2019.02277)
Supplement: Supplementary file 1 [file Table_1.DOC]

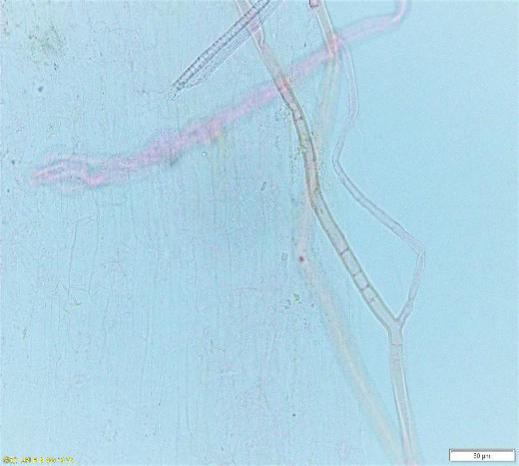


**50μm**


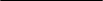


**G**

**Hy**


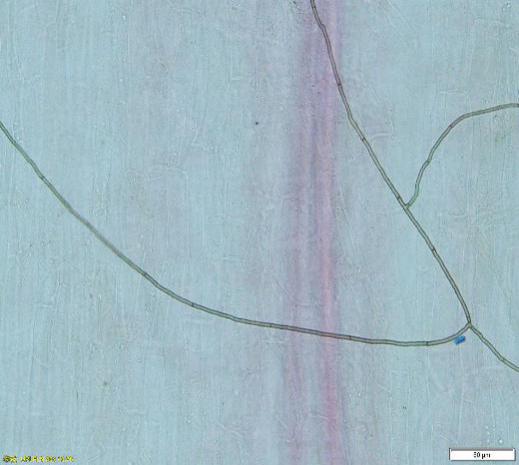


**50μm**


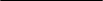


**Hy**

**E**


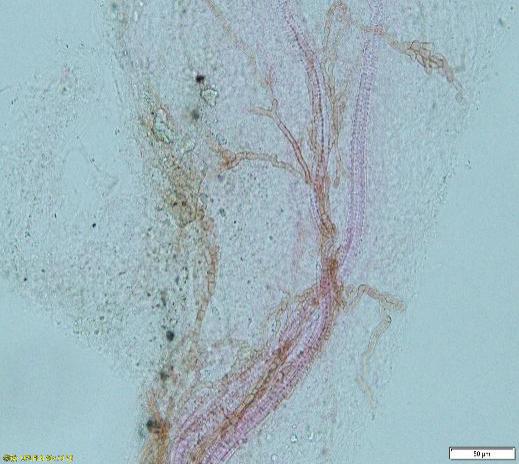

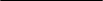


**50μm**

**Hy**

**C**


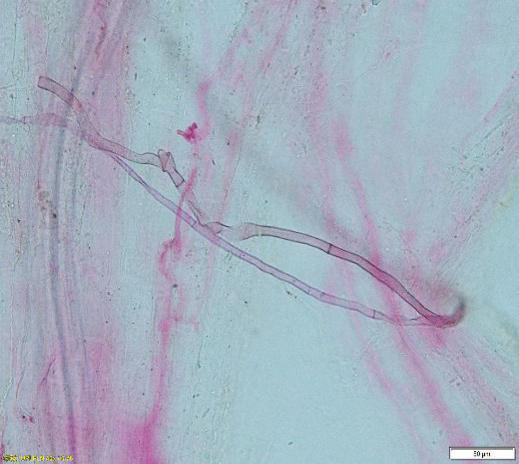


**50μm**

**Hy**

**A**


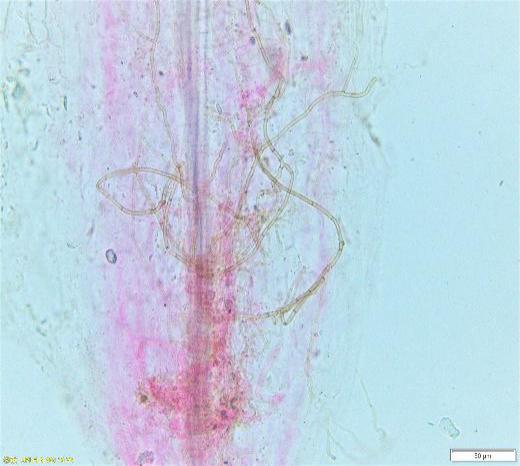

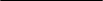


**50μm**

**Hy**

**F**


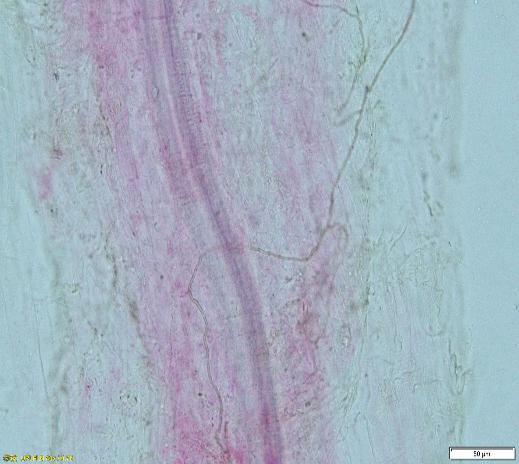

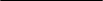


**50μm**

**Hy**

**D**


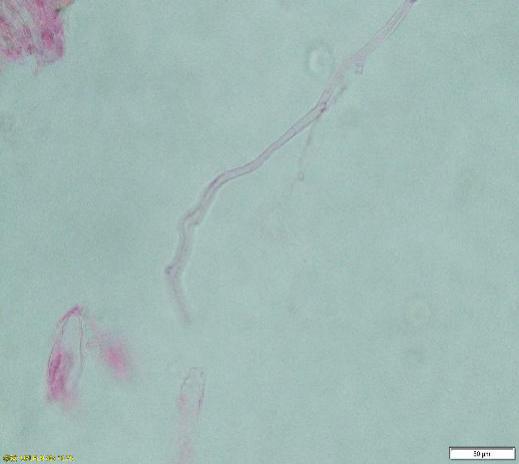


**50μm**


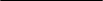


**Hy**

**B**


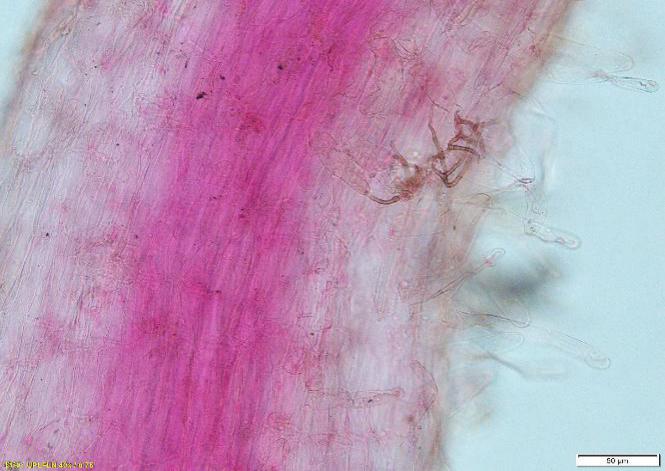


**50μm**


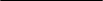


**Hy**

**H**

**Figure S1** Colonization of dark septate endophyte (DSE) strains in the roots of

liquorice plant three months after inoculation. A, C, E and G indicate roots of non-inculated and inoculated with *A. vagum*, *P. putaminum* and *F. acuminatum* under normal water condition; B, D, F and H indicate roots of non-inculated and inoculated with *A. vagum*, *P. putaminum* and *F. acuminatum* under drought stress, respectively. Arrows indicate: H, DSE hyphae.


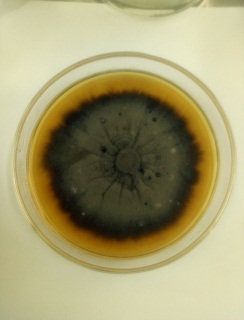


**B**


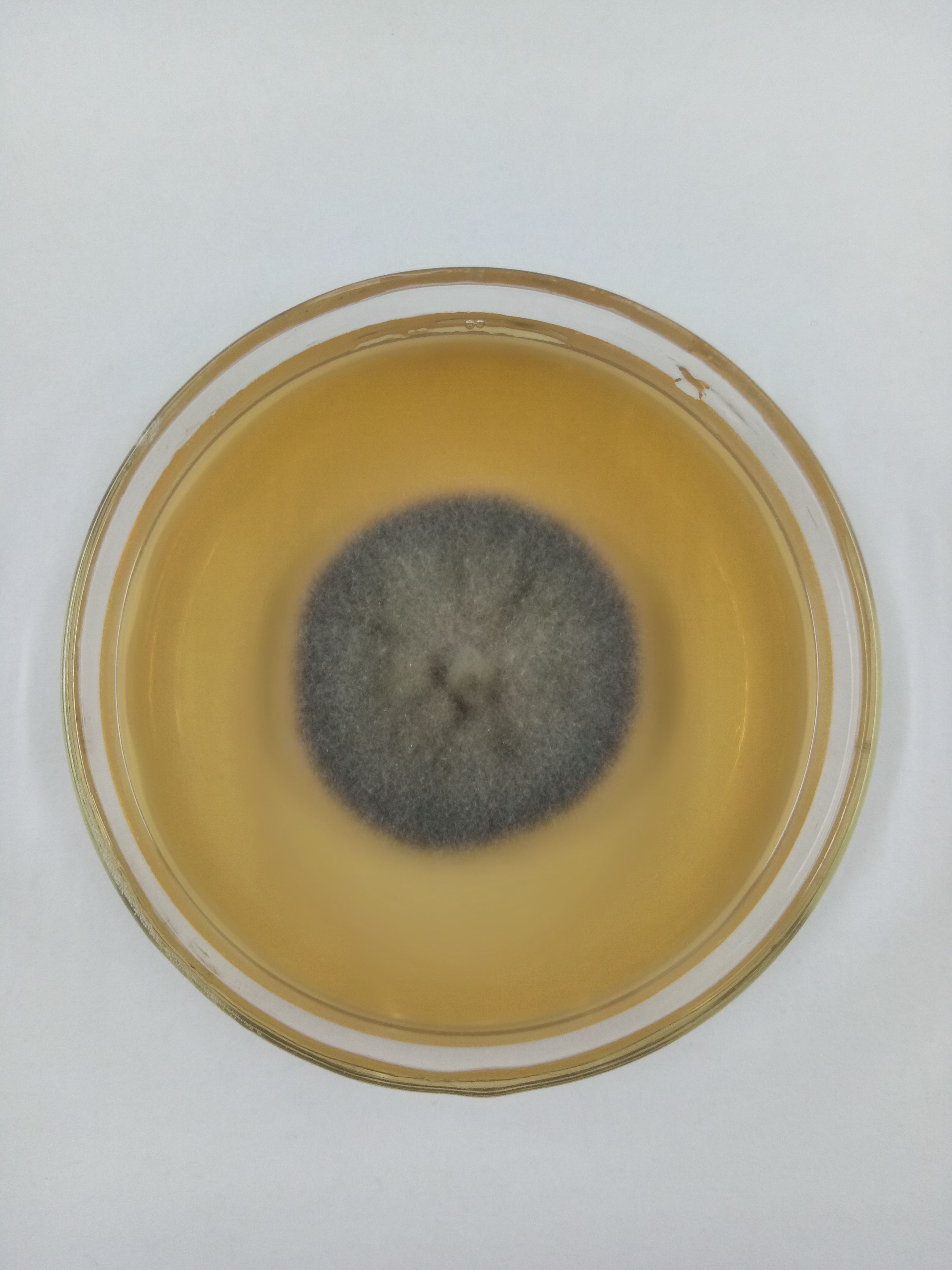


**A**


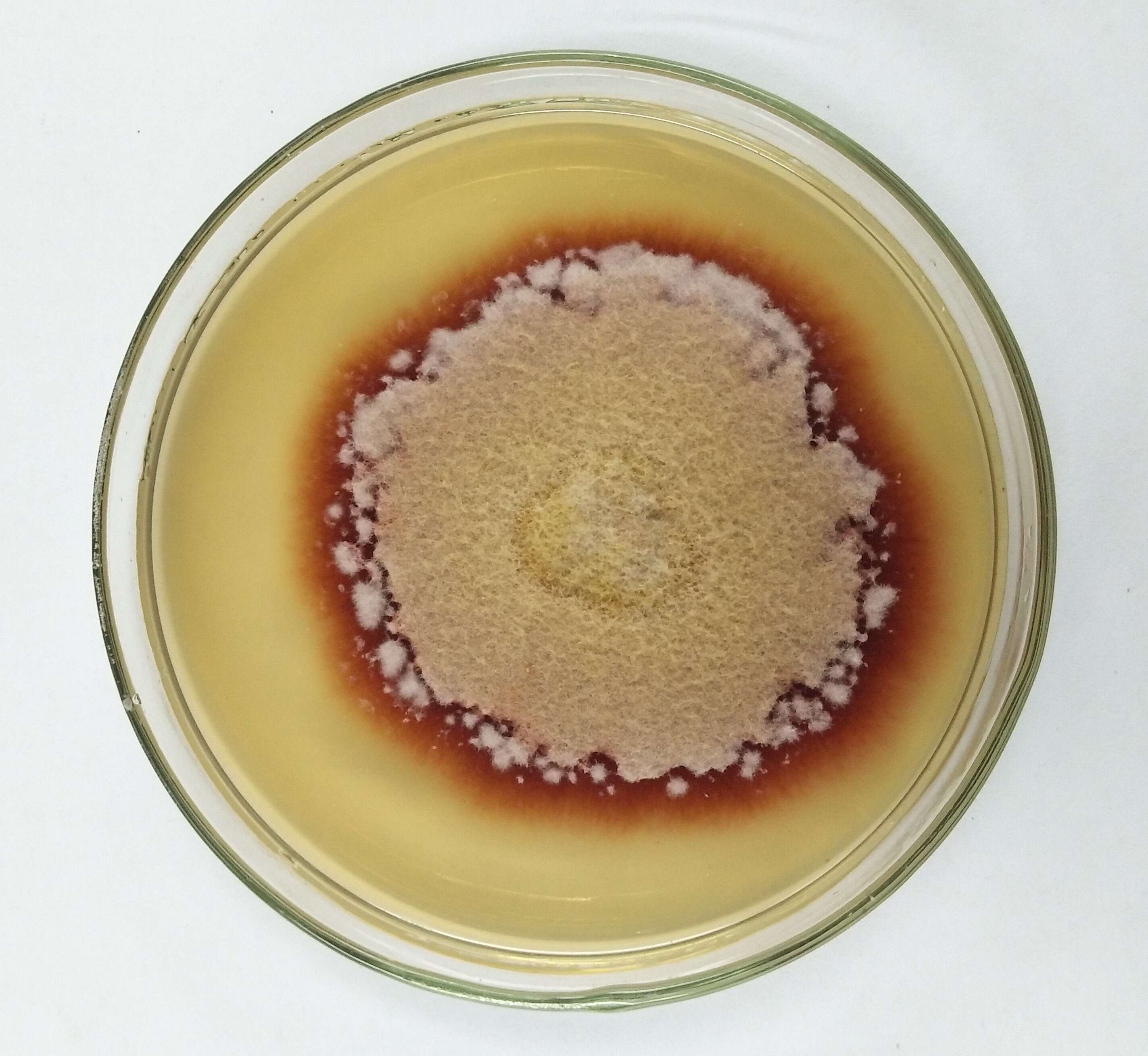


**C**


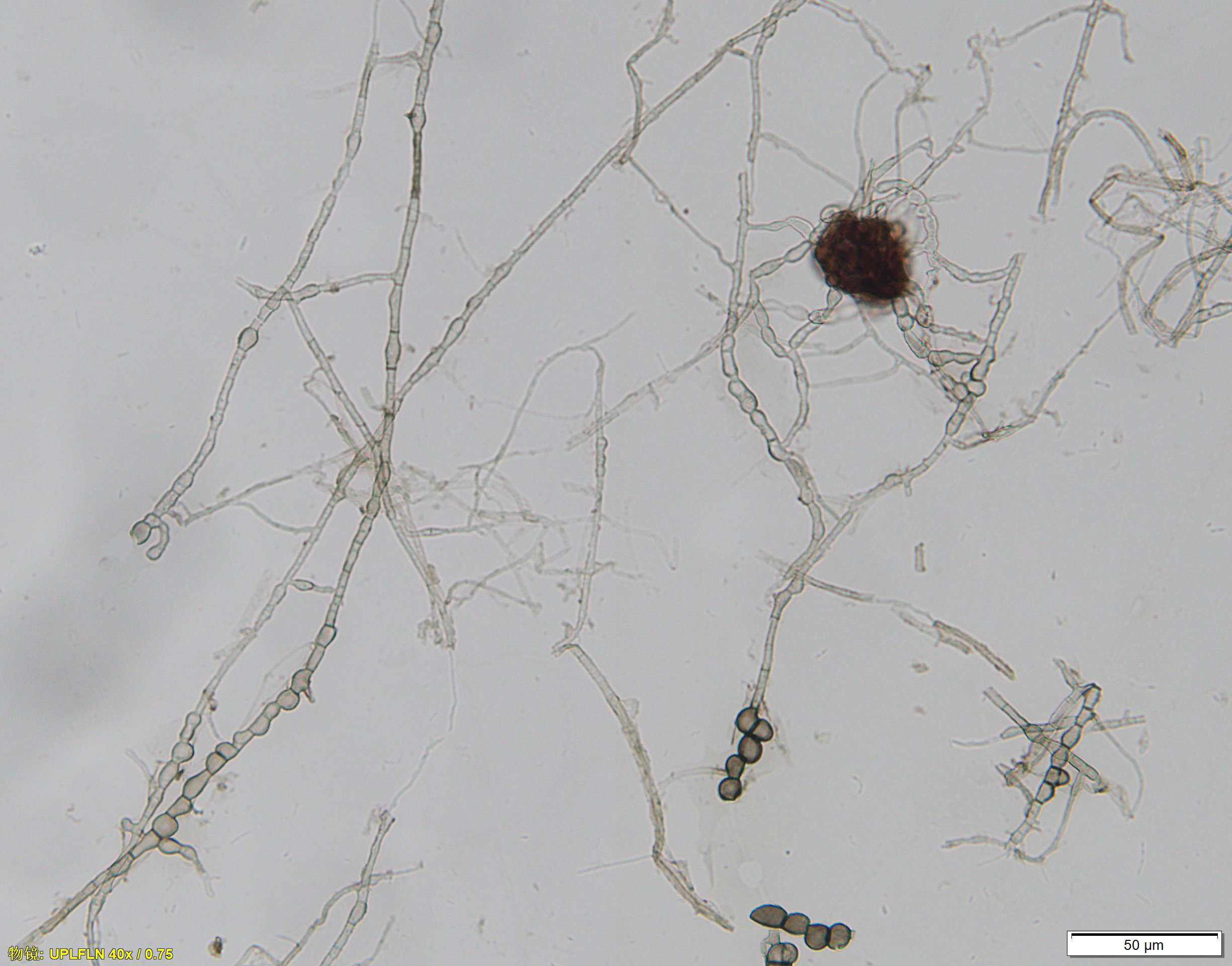


**a**

**Hy**

**S**

**50μm**


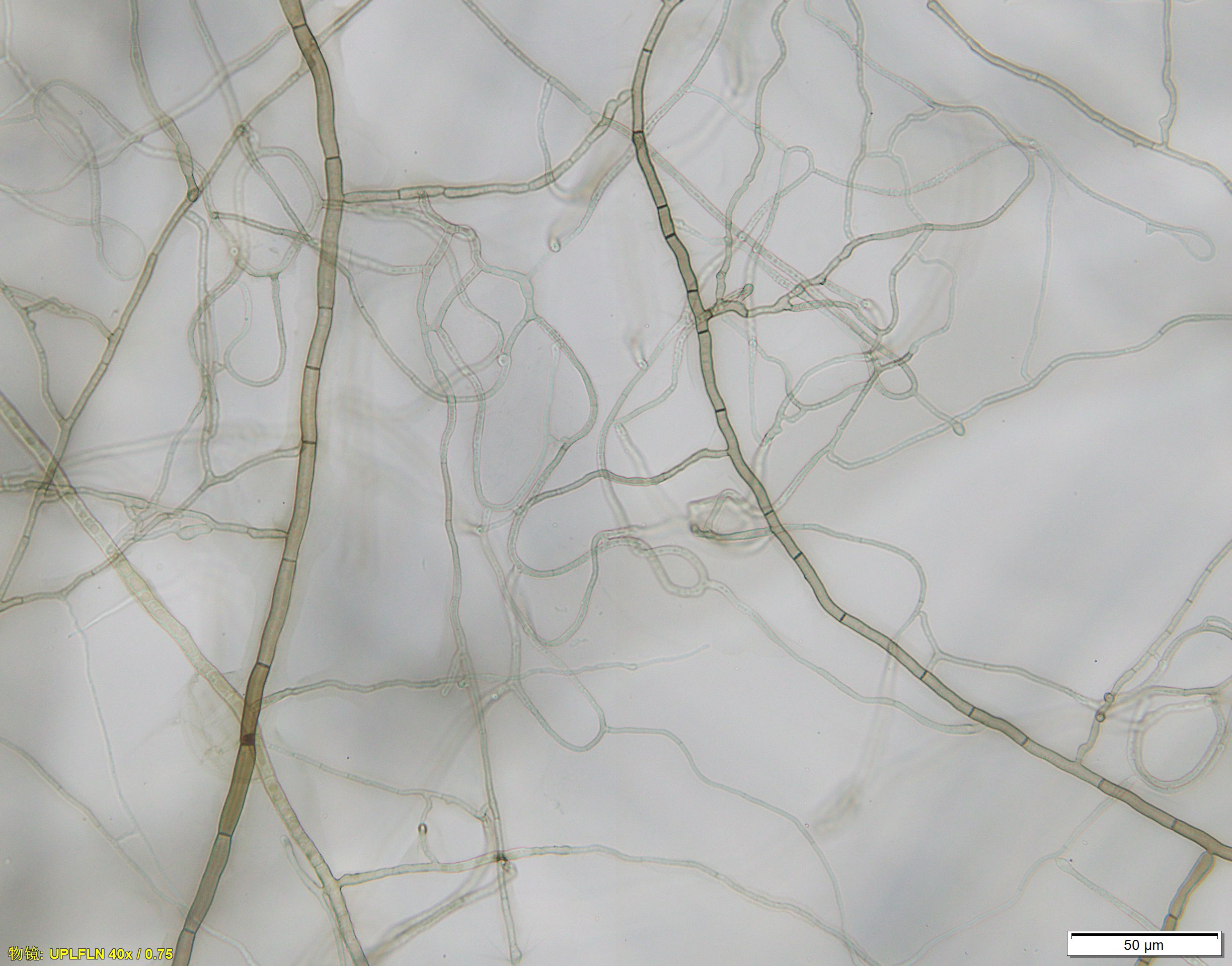


**b**

**Hy**

**50μm**


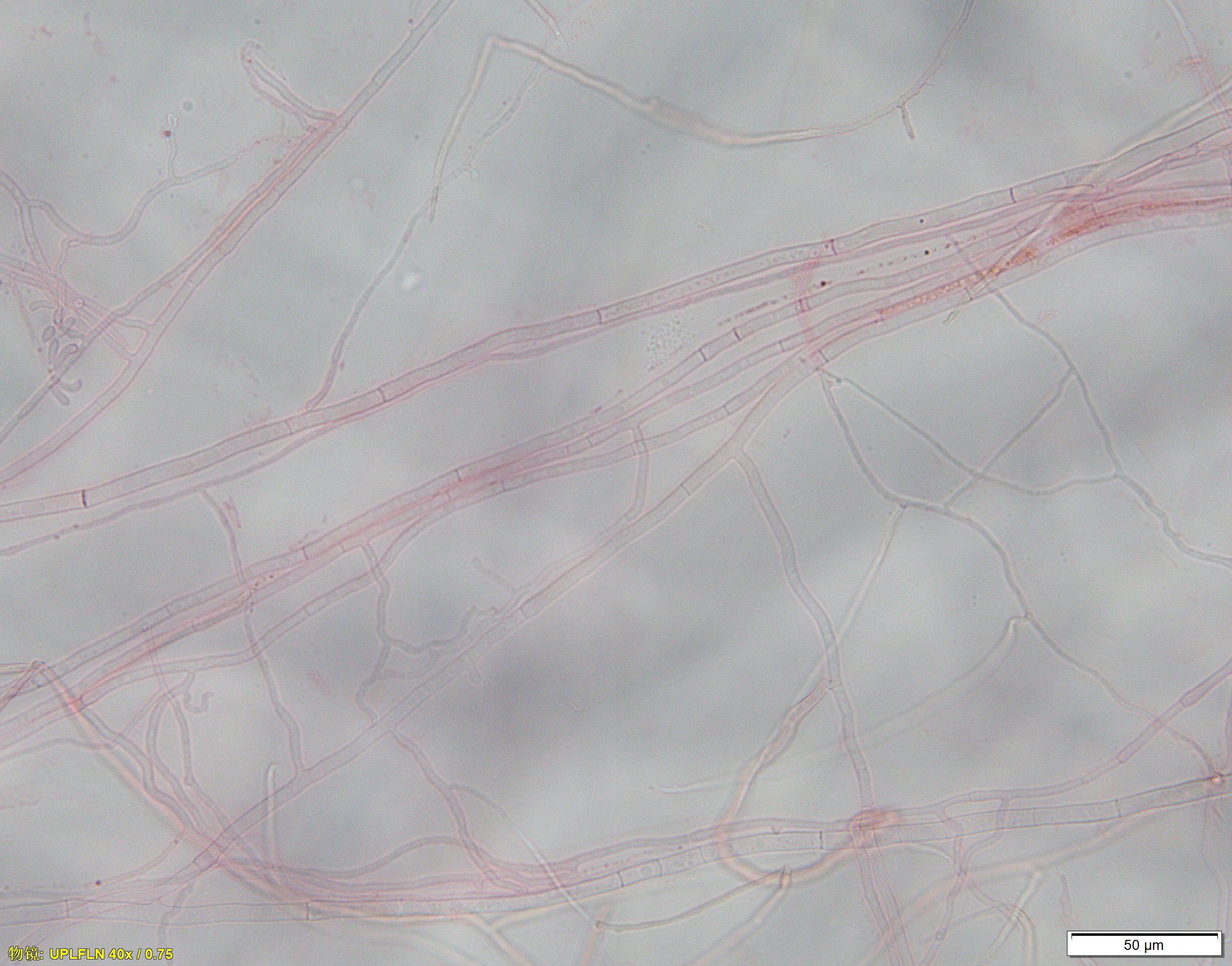


**Hy**

**c**

**50μm**

**Figure S2** Colonies of endophytic fungi isolated from the roots of liquorice plant three months after inoculation (A, B, C). Microscopic morphology of endophytic fungi (a, b, c). A, a: *A.vagum*; B, b: *P. putaminum*; C, c: *F. acuminatum* Arrows indicate: Hy, DSE hyphae; S, DSE spores.

**Table S1 HPLC mobile phase and** [**gradient elution**](http://dict.cnki.net/dict_result.aspx?searchword=梯度洗脱条件&tjType=sentence&style=&t=condition+of+gradient+elution)**.**

| Time (min) | A (acetonitrile) | B (deionized water : phosphoric acid) |
| --- | --- | --- |
| 0.0 | 14% | 86% |
| 10.0 | 23% | 77% |
| 24.0 | 30% | 70% |
| 30.0 | 34% | 66% |
| 35.0 | 36% | 64% |
| 42.0 | 42% | 58% |
| 48.0 | 51% | 49% |
| 60.0 | 14% | 86% |
